# A qualitative systematic review on the lived experience of men in nursing

**DOI:** 10.1002/nop2.1269

**Published:** 2022-06-05

**Authors:** Xiaochen Lyu, Thitinut Akkadechanunt, Pratum Soivong, Phanida Juntasopeepun, Ratanawadee Chontawan

**Affiliations:** ^1^ The First Affiliated Hospital of Wannan Medical College Wuhu China; ^2^ Faculty of Nursing Chiang Mai University Chiang Mai Thailand

**Keywords:** lived experience, male nurses, men in nursing, qualitative research, systematic review

## Abstract

**Aim:**

This qualitative systematic review was conducted to describe the lived experience of men in nursing.

**Design:**

A systematic review of qualitative studies.

**Methods:**

Five databases (Scopus, CINAHL, MEDLINE, PsycINFO and Embase) were systematically searched. The PRISMA guideline was used for reporting the literature search in different phases, and the Critical Appraisal Skills Program, a qualitative research checklist, was used to evaluate the studies that met the inclusion criteria. Thomas and Harden's thematic analysis approach for qualitative research was used for data synthesis.

**Results:**

Six qualitative studies were included. Five analytical themes related to lived experiences of men in nursing emerged: value in nursing, the double‐edged sword of gender, being accepted in the nursing profession, attractions of nursing and coping strategies.

## 1 INTRODUCTION

With the growth of a diversified population, the demand for health care has become more complex. It is crucial to ensure that there is a sufficient and diverse supply of registered nurses (RNs) to meet the healthcare needs of people nationally and worldwide (Blair, 2016; Douglas et al., 2014). Men in nursing, although a minority group in most countries, are important for health care as a whole. Men in nursing could provide diverse forms of nursing care to the nursing profession, especially for male patients (Kim & Shim, 2018). Sometimes patients prefer nurses of a certain gender because they may feel more comfortable and their privacy is protected, especially when intimate care is provided (Seal & Wiske, 2018). In addition, the physical strength of men could deliver some benefit in certain cases for healthcare institutions. For instance, the physical strength of men can provide safe nursing care for patients in the department of psychiatry, and they can easily move patients and heavy medical equipment in the orthopaedics department (Halcomb et al., 2012). Thus, recruitment and retention of men in the nursing profession are considered important components that will increase the supply of nurses in the workforce and provide care for the diverse population.

Although small increases in their representation have occurred in the last several years, the number of men in nursing is still grossly disproportionate compared to that of women nurses. According to the United States Bureau of Labor Statistics (2019), around 12% of registered nurses in the United States are men. A similar situation has occurred in other countries around the globe: 2.2% in China (National Bureau of Statistics of China, 2018), 9% in Canada (Canadian Nurses Association, 2019), 8% in New Zealand (New Zealand Nurses Organization, 2018), 10.8% in the United Kingdom (Nursing and Midwifery Council of UK, 2021) and 11.75% in Australia (Nursing and Midwifery Board of Australia, 2017).

## 2 BACKGROUND

With the increasing number of research studies on men in nursing, it is time to evaluate and summarize the existing literature in order to gain a deeper understanding of the lived experience among men in the nursing profession. Through a literature review, we found that there is a variety of lived experiences of men in nursing. Several studies reported that men in nursing always experience isolation and discrimination from female colleagues and patients in clinical environments (Adeyemi‐Adelanwa et al., 2016; Gedzyk‐Nieman & Svoboda, 2019; Kronsberg et al., 2018). In addition, some previous studies reported that men continue to encounter many stereotypical social images, such as gender barriers (Coleman, 2008; Keogh & O'Lynn, 2007; Rowlinson, 2013), lower masculinity (Harding, 2007; Saleh et al., 2020) and role strain (Carte & Williams, 2017) in the nursing profession. On the contrary, another strand of the literature found that men in nursing had a positive lived experience as a minority group. Men in nursing are appreciated and preferred by patients and other healthcare workers compared with their women peers in clinical nursing as providers of unique and quality nursing care (Achora, 2016; Carte & Williams, 2017).

The unique healthcare and education backgrounds, along with the distinct cultural contexts, could lead to distinctive lived experiences of men in nursing in different countries. Thus, it is necessary to proceed with a systematic review of the lived experience of men in nursing worldwide based on previous studies, thereby providing a holistic understanding of lived experiences among men in the nursing profession. This systematic review aims to analyse and criticize the current literature on the lived experience of men in nursing and attempts to present a cogent understanding of their lived experiences. Based on qualitative evidence, it would help healthcare policymakers develop applicable and transferable strategies for improving their experience in the nursing profession and provide further recommendations for formulating relevant policies and procedures.

**Research question**

What are the lived experiences of men in nursing?

## 3 THE STUDY

### 3.1 Design

A systematic review and synthesis of qualitative studies were conducted. The thematic analysis approach of Thomas and Harden (2008) that is used in this inquiry is a strategy for identifying, analysing and synthesizing qualitative research. This method allows the researcher to “go beyond” the conclusion of the original analyses to formulate a new interpretation of the phenomenon (Thomas & Harden, 2008).

### 3.2 Search methods

The guideline of PRISMA was used to conduct this qualitative systematic review. The five databases of Scopus, CINAHL, MEDLINE, PsycINFO and Embase were searched, in English, using the following search terms: nursing, male nurses, male registered nurses, men in nursing, lived experiences and nursing profession from January 2000 to February 2021. Initially, through a systematic search of the literature based on the five databases, relevant studies were not found, particularly those focussed on the lived experience of men in nursing prior to the year 2000. Next, the researchers contacted experts in the subject area through email to obtain grey literature or unpublished sources of evidence. Finally, we searched the reference list of related studies to complete an exhaustive search of eligible papers. The inclusion criteria and exclusion criteria can be seen in Table 1.

**TABLE 1:** Review inclusion and exclusion criteria

Inclusion criteria	Exclusion criteria
Papers are written in English between January 2000 and February 2021. Papers focussed on: Adopted a qualitative methodology. Explored the lived experience of male nurses. Included participants who had at least 1‐year experience in clinical nursing. Papers were full peer‐reviewed studies.	Papers not written in English. Papers were quantitative studies. The research participants were male nursing students and novice male nurses with less than 1 year of clinical experience.

### 3.3 Search outcomes

**FIGURE 1:**
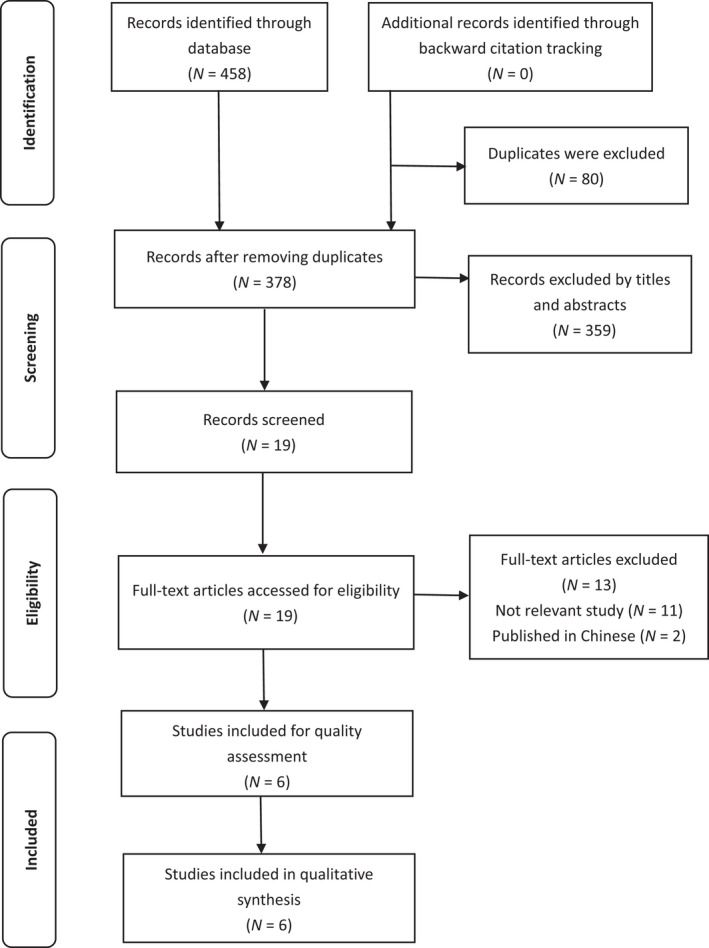
PRISMA flow diagram

The search resulted in 458 potential studies. Based on inclusion and exclusion criteria, two authors reviewed the title and abstract independently. When they had disagreements on articles, a third author would join the discussion until a consensus was reached. Subsequently, four articles that met the inclusion criteria were selected. The authors found three unpublished Ph.D. theses related to our research and obtained the full text after contacting their authors by email. Lastly, after reviewing these three relevant articles, one article did not meet the criteria of inclusion; therefore, it was excluded. Finally, six articles were included in this study. Figure 1 outlines the PRISMA flow diagram.

### 3.4 Quality appraisal

The Critical Appraisal Skills Program (CASP) Checklist for Qualitative Research (Critical Appraisal Skills Program, 2019) was used to assess the quality of the six selected studies. CASP is a widely used critical assessment tool for assessing the quality of reports of research outcomes including qualitative literature reviews. In all of the included studies, a quality assessment of each study was undertaken by two reviewers independently using the CASP for qualitative research to ensure ethical standards and methodological rigour were met. The outcomes of the quality assessment for the six studies are shown in Table 2.

**TABLE 2:** Quality assessment

Protocol statement	CASP questions	Paper, author (data)					
		Rowlinson (2013)	Achora (2016)	Blair (2016)	Frimpong (2016)	Saleh et al. (2020)	Smith et al. (2020)
Adopted an appropriate method and design to meet the aims of the study	Was there a clear statement of the aims of the research?	Yes	Yes	Yes	Yes	Yes	Yes
	Is a qualitative methodology appropriate?	Yes	Yes	Yes	Yes	Yes	Yes
	Was the research design appropriate to address the aims of the research?	Yes	Yes	Yes	Yes	Yes	Yes
Used a suitable data collection strategy	Was the recruitment strategy appropriate for the aims of the research?	Not known	Yes	Yes	Yes	Yes	Yes
	Was the data collected in a way that addressed the research issue?	Yes	Yes	Yes	Yes	Yes	Yes
	Has the relationship between researcher and participants been adequately considered?	Not known	Yes	Yes	Yes	Yes	Yes
Included pertinent methods of data analysis	Was the data analysis sufficiently rigorous?	Yes	Yes	Yes	Yes	Yes	Yes
Drew conclusions and interpretations that reflected the findings of the study	Is there a clear statement of findings?	Yes	Yes	Yes	Yes	Yes	Yes
	How valuable is the research?	Valuable	Valuable	Valuable	Valuable	Valuable	Valuable
Obtained ethical approval	Have ethical issues been taken into consideration?	Yes	Yes	Yes	Yes	Yes	Yes

### 3.5 Data extraction and synthesis

Using a predesigned form, the relevant data were extracted by two authors, including descriptive data (demographic details, research objectives, study design and participants) and analytical data (results/themes). All text labelled as “findings” or “results” should be extracted (Thomas & Harden, 2008). The data analysis and synthesis were conducted using a thematic analysis approach for qualitative research developed by Thomas and Harden (2008). In the first step, two authors coded the text “line‐by‐line,” independently. Secondly, a “descriptive theme” was created to summarize the meaning of the initial code group. Finally, the “analytical themes” were generated.

### 3.6 Ethics

The ethics board approval and acquisition of participants' informed consent were not required as this study was performed on published data.

## 4 RESULTS

Based on the aims of this review about the lived experience of men in nursing, six studies were identified which came from four countries: the United States, the United Kingdom, Uganda, and Jordan. These studies generated findings from 72 male registered clinical nurses. Sample sizes ranged from 1 to 22, and the participants ranged in age from 27 to 65 years old. All of the participants had at least 1 year's experience in a clinical setting. Table 3 presents the characteristics of all of the studies. Finally, five analytical themes were identified as follows: (1) value in nursing, (2) the double‐edged sword of gender, (3) being accepted in the nursing profession, (4) attractions of nursing, and (5) coping strategies. Table 4 outlines the themes in each article.

**TABLE 3:** Bibliography of included studies

Author, year, origin	Aim of study	Study design	Data collection and analysis	Sampling (*n*)	Demographic details	Main findings
Rowlinson (2013), United Kingdom	To explore the lived experience of nursing from both male and female perspectives	Qualitative, hermeneutic phenomenological approach	Semi‐structured interviews, 1 hr. Interpretative phenomenological approach	Purposive (1)	Participant working in clinical practice and was in his late 20s and had been qualified for more than 6 years	Intersubjectivity Career versus vocation Gender stereotyping
Achora (2016), Uganda	To explore and describe the lived experiences of male nurses in a Ugandan hospital	Qualitative, descriptive	Interviews, 60 min, guided phenomenological reflection, Colaizzi's phenomenological approach	Purposive (11)	Participants' ages ranged from 27 to 42 years with nursing experience of 4 to 11 years	Being appreciated as expressing unique nursing care Being misunderstood as practitioners of other disciplines Being maltreated by colleagues of the profession and other healthcare workers
Blair (2016), United States	To explore and elucidate the everyday lived experience of male registered nurses in the workforce	Qualitative, hermeneutic (interpretive‐descriptive) phenomenological approach	Semi‐structured interviews, 60 min, Qualitative content analysis	Purposive (17)	Participants ages ranged from 30 to 65 years with nursing experience of 2 to 40 years	Motivating factors (job opportunities, job flexibility, financial stability) Breaking gender barriers (moving beyond gender barriers, making a difference, caring for female patients) Gendering (preferential privileges, maintaining masculinity)
Frimpong (2016), United States	To explore the lived professional experiences of male nurses and understand the factors which impacted the professional experiences of male nurses	Qualitative, phenomenological lifeworld	In‐depth interviews (60 min), focus group (90 min), and participants' diaries, Inductive approach and narrative analysis	Purposive (10)	Participants ages ranged from 38 to 54 years with nursing experience of 4 to 13 years	Separateness Discrimination Job security and benefits Career opportunities Gender‐based stereotypes Caring through spirituality Glass (d) escalator
Saleh et al. (2020), Jordan	To explore the Jordanian male nurses' experiences of their careers in their Arabic community	Qualitative, hermeneutic phenomenological methodology	Focus group, 75–90 min, Van Manen's hermeneutic approach and Phenomenology of Practice	Purposive (22)	Participants ranged in age from 27 to 45, with 5 to 15 years of experience	Personal gains (privileged to ease patient's suffering; privileged to fulfil our spiritual needs; job security and economic stability) Masculinity (stereotype characteristics; endurance) Cultural influences (positive aspects of social milieu; Negative aspects of social milieu)
Smith et al. (2020), United States	To investigate the lived experiences of male nurses in today's healthcare environment to understand the persistently low numbers of men in nursing	Qualitative, interpretive description methodology	Semi‐structured interviews: Group interview, 60–180 min; Individual interviews, 50–90 min, Inductive approach and discourse and narrative analysis	Purposive (11)	Participants ages ranged from 30 to 64 years with nursing experience of 2 to 41 years	Role expectations (societal views, professional acceptance, patient/family feelings) Workplace relations (being male in a female environment, social cliques and peer support)

**TABLE 4:** Presence of themes in each paper

Theme	Category	Rowlinson (2013)	Achora (2016)	Blair (2016)	Frimpong (2016)	Saleh et al. (2020)	Smith et al. (2020)
**Value in nursing**		√		√	√	√	
Meaningful to patients	Easing patients' suffering Patient‐centred	√		√		√	
Fulfils spiritual needs	Higher‐level communication with patients Caring through spirituality				√	√	
**The double‐edged sword of gender**		√	√	√	√	√	√
Gender disadvantages	Discrimination Separateness Being maltreated Being misunderstood	√	√		√	√	√
Gender advantages	Endurance Preferential treatment Glass escalator Easy to stand out	√		√	√	√	
**Being accepted in the nursing profession**			√			√	√
	Being accepted by patients		√			√	√
	Being accepted by female peers					√	√
**Attractions of nursing**		√		√	√	√	
	Career opportunities	√		√	√	√	
	Financial stability			√	√	√	
	Job flexibility			√	√		
**Coping strategies**			√	√			√
Creating a role model of men in nursing	Making a difference Moving beyond barriers		√	√			√
Adapting to a women‐dominated clinical setting	Being male in a female environment Caring for female patients			√			√

### 4.1 Value in nursing

Understanding the meaning of the word “value” is a prerequisite for defining the value of nursing. A value is an abstract representation of what is right, important, worthwhile or desirable (Black, 2019). In this study, values reflect what nurses considered desirable and included the subjective assignment of value to their behaviours. Although men in nursing do not realize it, values help them make small daily choices and important life decisions. Just as beliefs influence nursing practice, values also influence a nurse's professional practice, often unconsciously (Black, 2019). Two analytical sub‐themes were identified about the value of nursing: “meaningful to patients” and “fulfills spiritual needs.”

#### 4.1.1 Meaningful to patients

As nursing is a “caring and helping for people” profession, nurses are given the privileged responsibility of caring for the sick, injured and dying. That is, nurses have the opportunity to make a difference in the lives of others by giving care to others in their time of need, just as you would want someone to do for you if you were in their place. The participants explained that nursing is a worthwhile job because it provides care and promoted well‐being for patients (Rowlinson, 2013):

I realized that nursing is a worthwhile job with patients when I provide caring for them. (Participant MN, Rowlinson, 2013, p. 220)

This type of lived experience exists for men in nursing in the Arab world as well (Saleh et al., 2020). As described by a participant, nursing is a humanistic profession, and the basic principle of nursing is helping others, thereby obtaining emotional growth:

Nursing gives me the chance to ease the pain and suffering of cancer patients (Participant FG3:P1, Saleh et al., 2020, p. 317)

As clinical nurses, they reported being frequently involved in some of life's most meaningful moments for patients. They especially perceived happiness and pleasure when the patients get better and recover under their care. Most participants explained that the nature of helping is a really meaningful thing for both patients and themselves (Saleh et al., 2020):

In ICU, I was left with my heart full of happiness when my patient's suffering was reduced, and he was extubated and improved. (Participant FG4:P1, Saleh et al., 2020, p. 317)

#### 4.1.2 Fulfils spiritual needs

Spirituality is rooted in trying to understand the meaning of life, which resides within the individual and what they personally believe. Nursing was described as a “call to duty from above” (Frimpong, 2016) by participants, and they also believed that it provided a chance for them to be close to God (Saleh et al., 2020). This reflected that men in nursing acquire the spiritual need of finding meaning and purpose in life through nursing care:

When I pray with patients, I comfort them with the words of God to believe again and they recover more quickly to go home and never to return to the hospital. (Participant Kwadwo, Frimpong, 2016, p. 89)

Spirituality plays a very important role in some countries with religious beliefs because it is embedded in the fabric of society and culture (Frimpong, 2016; Saleh et al., 2020). The participants consistently claimed faith in the healing power of prayer and its significant influence on their careers. Participants suggested that nurses should not deny spiritual practices such as prayer in their nursing care but should be encouraged to pray together with patients to alleviate the disease.

I pray to commit my patients into the Lord's hands at the start of my shift for healing… I ask patients to hold my hands in faith and we pray together for healing, and they begin to recover. (Participant Kwaku, Frimpong, 2016, p. 88)

Additionally, serving patients and alleviating sickness represents an aspect of spirituality by finding the meaning and purpose of life. Almost all participants indicated that becoming more caring and humanistic was a positive feature of being a nurse (Frimpong, 2016; Saleh et al., 2020). Meanwhile, they received spiritual satisfaction from their clinical practice:

My daily interactions with patients forever strengthen my faith as a Christian and they give me a reason to be a male nurse. (Participant Kwaku, Frimpong, 2016, p. 89)

### 4.2 The double‐edged sword of gender

Martin (2003) mentioned that women and men used a two‐sided dynamic of gendering practices and practising of gender to construct each other at work, and this dynamic significantly affects both men's and women's work experiences. Martin (2003) defines gendering practices as “the literal activities of gender, physical and narrative – the doing, displaying, asserting, narrating, performing, mobilizing, and maneuvering” (p. 354). Gendering practices in the workplace can influence people negatively or positively. They can create opportunities and inequalities. Two analytical sub‐themes were identified about the double‐edged sword of gender: “gender disadvantages” and “gender advantages.”

#### 4.2.1 Gender disadvantages

Persistent gender stereotypes of men in nursing were held by patients, women nurses and even other professionals in most studies (Achora, 2016; Frimpong, 2016; Rowlinson, 2013; Smith et al., 2020). Although more and more men have chosen the nursing profession in recent years, the dominance of women remains unchanged. Men are in no way barred from entering this line of work, but there is various gender stereotyping they face when pursuing this predominantly women's field.

It's still seen as a women's field or a women's role. (Participant MN, Rowlinson, 2013, p. 221)

There are many women nurses who also hold gender‐based stereotypes of men in nursing in a clinical setting. The participants explained that they are always considered stronger and better able to do physical work than women nurse peers (Achora, 2016; Frimpong, 2016). Thus, men in nursing were always automatically assigned to do difficult procedures in the unit, and they were also often asked to help women nurses with the physical work.

……Male nurses are assigned the most tiresome and difficult tasks by in‐charges; such tasks are not given to female nurses. (Participant 11, Achora, 2016, p. 26)

In obstetrics and gynaecology settings, gender stereotypes are more obvious and challenging especially when “sensitive procedure” and “intimate care” were involved (Achora, 2016; Saleh et al., 2020). In such situations, the legitimacy of the nursing care offered by men was questioned:

……During providing home care, I was attacked by a furious husband who asked why I continue to follow his wife at home. He asked me to leave his home immediately saying I seem to be in love with his wife. (Participant 1, Achora, 2016, p. 26)

Similarly, gender‐based stereotypes also exist in social life. Both men and women nurses are often described as subservient to doctors on television and in films, suggesting nursing is a lesser profession (Smith et al., 2020). As a man in the nursing profession, it is hard to find a girlfriend. Because many people refused to marry their daughters to men in nursing (Saleh et al., 2020). Men in nursing said that this is a socially limiting issue.

When I plan to get married, I have limited choices; people do not prefer to marry their daughters to a male nurse, especially if she is a physician or an engineer. (Participant FG4:P3, Saleh et al., 2020, p. 319)

#### 4.2.2 Gender advantages

Conversely, gender brings some advantages for men in nursing as well. The participants shared their experiences on how their super‐resilience and endurance afforded them preferences and privileges even though women nurses disproportionately outnumber them. Most of the participants stated that male physicians typically treat them with more respect and value their opinions more than their women co‐workers do (Blair, 2016;Saleh et al., 2020; Smith et al., 2020). In addition, the participants also supported the argument that men in nursing have easier promotion prospects than their women counterparts in the clinical setting (Blair, 2016; Saleh et al., 2020):

My boss latched onto me much closer than she would with other female nurses that I've started with. Within a given year, I probably received two or three promotions over the females that have similar qualifications. (Participant Bonah, Blair, 2016, p. 120)

Most of the participants believed due to gender advantages, men in nursing gravitated to intensive care units, emergency departments or operating rooms because there is more autonomy, more technical equipment and collaboration in the decision‐making of patients (Blair, 2016):

I think it's more of like, they like the adrenaline, they like the high, fast‐paced and stressful environment. It seemed to be something that most men like. Because us males, we do love challenges, okay. (Participant Nate, Blair, 2016, p. 120)

Besides, the participants explained that they also have super‐resilience and endurance (Saleh et al., 2020); hence, they are always seen to be more emotionally stable in different situations than women nurses, such as when dealing with contingencies in clinical nursing (Blair, 2016):

A female nurse usually cannot control her feelings and starts to cry and cannot continue with us. (Participant FG3:P3, Saleh et al., 2020, p. 318)

### 4.3 Being accepted in the nursing profession

Participants had many years of nursing experience and diverse clinical backgrounds. They discussed acceptance in a predominantly women's profession and how they are often preferred over their women counterparts as providers of unique, quality nursing care by patients (Achora, 2016; Smith et al., 2020). Men in nursing are always considered as being approachable and trustworthy by patients:

……In most cases male nurses are more approachable than the female nurse, and male nurses like to spend more time with the patients; hence, they are popular with the patients. (Participant 4, Achora, 2016, p. 26)

Especially when it comes to invasive treatment, patients and their families prefer to be served by male nurses because, as men, they were perceived as more competent to do this. In general, men in nursing were frequently well received by their patients (Saleh et al., 2020):

I work in a cardiac center, and patients prefer us to female nurses to walk them out of their beds. Also, patients prefer us to female nurses to insert their cannulas. (Participant FG2:P5, Saleh et al., 2020, p. 318)

Furthermore, most participants discussed the acceptance and support received from their women nurse peers (Saleh et al., 2020; Smith et al., 2020). Participants said a deep sense of gratitude towards their women nurse peers and attributed much of their success to the support they received from them. Some participants explained that their women nurse peers provided assistance to them when “intimate care” with women patients was involved; women nurse peers always spoke highly of the service provided by male nurses to patients during bedside reports in OB & GYN settings (Saleh et al., 2020; Smith et al., 2020). The acceptance and support that men in nursing received from their women nurse peers had a profound effect on their patients and were integral to their success:

I owe much, if not most of my success to the wonderful female nurses I have worked with. I've never been made to feel like a male nurse. Just a nurse. (Participant, Smith et al., 2020, p. 1214)

### 4.4 Attractions of nursing

Although the number of men in nursing is disproportionate, some men still choose nursing because it can provide many job opportunities for them. The nursing field offers many speciality job opportunities for men, such as emergency or trauma, psychiatry, oncology nursing and even nursing education and nursing administration (Blair, 2016; Frimpong, 2016). Nursing is not a one‐size‐fits‐all profession; for both men and women nurses, the wide array of job opportunities makes it possible to pursue one's specific areas of interest in the field.

I chose nursing administration when I started my career but with my experience and interest in teaching, I'm pursuing a new opportunity in nursing education to become a full‐time nurse educator. (Participant Nkrumah, Frimpong, 2016, p. 83)

The participants explained that security and stability are other motivations for choosing nursing, and it shows signs of increasing. Nursing has long been considered a reliable career option because of the attractive benefits it offers both in service and retirement (Blair, 2016; Saleh et al., 2020). It is also not uncommon for novice nurses to be offered big bonuses in the nursing profession (Frimpong, 2016). Even in today's shaky economy, nursing is a reliable and financially savvy career choice for men:

I can get a good salary in nursing, and if I were in another job, I believe it would be hard for me to secure a job with such a good salary. (Participant FG2:P5, Saleh et al., 2020, p. 317)

In addition, the majority of participants said that, for whatever reason, all nurses enjoy a benefit that most professional occupations cannot offer: work flexibility. With the growing emphasis on work‐life balance, nursing has been lauded as one of the few career choices that offer flexible options throughout a lifetime (Blair, 2016; Frimpong, 2016):

… Um, nursing is a lot more flexible. I wanted to spend time with my son so that was a big, um, part of it all. Then I looked at nursing and I, it just, it made the most sense for me. You know money‐wise, time‐wise, flexibility‐wise, for me it worked out fine. (Participant Kent, Blair, 2016, p. 99)

### 4.5 Coping strategies

Most participants have encountered a variety of gender stereotypes and gender bias in the nursing profession as mentioned above. At the same time, they also shared coping strategies for dealing with these gender stereotypes and biases, in order to change an unfavourable situation. Concerning coping strategies, two key sub‐themes were identified: “creating a role model of men in nursing” and “adapting to a women‐dominated clinical setting.”

#### 4.5.1 Creating a role model of men in nursing

Most participants said the opinion that men in nursing are not as publicly visible as women nurses or other healthcare professionals, especially with the lack of a public role model of men in nursing. They believed that the first step is to make the public aware of the existence of men in nursing and increase the public visibility of men in nursing which is a prerequisite for the acceptance of men in nursing by the public. (Blair, 2016; Smith et al., 2020). Meanwhile, most participants explained that they need to be proactive to publicly identify themselves as nurses and build a “superstar image” in as many public places as possible to increase visibility as men in nursing (Smith et al., 2020):

I think, the only way to do that is to be out in the public…They've never seen a male nurse before…I think, that we need to be out in the community showing our neighbors that I'm a male nurse. You may not know it, but I am. (Participant, Smith et al., 2020, p. 1214)

As participants shared their experiences, they stressed that as men in the nursing profession, they needed to be motivated and get involved in raising awareness about men in nursing (Blair, 2016; Smith et al., 2020). Moreover, men in nursing should set a role model for male nursing students who will be nurses in the future, which will raise the awareness that the nursing profession is gender diverse (Blair, 2016). Thus, it would further encourage the younger generation of men to see nursing as being appropriate for any gender, men or women, and that it is nothing to be embarrassed about:

Well, I really think that if we had more male educators, it would encourage more males in the profession, because as males you know, we always want to be looked at as a tough guy, the macho guy, so if we have more role models, more male educators, I think more of us would gravitate towards the field. (Participant John, Blair, 2016, p. 110)

#### 4.5.2 Adapting to a women‐dominated clinical setting

The participants explained that they needed to be prepared to adapt to a predominantly women's environment. Men in nursing agreed that adaption is necessary to avoid being isolated by female nurses and ensure survival in a profession dominated by women (Blair, 2016; Smith et al., 2020):

You learn to adapt to your environment and when your environment is many pretty women surrounding you, that's it. I adapted to the environment. I will survive. I couldn't survive if I didn't. (Participant, Smith et al., 2020, p. 1216)

In addition, the participants stated that they also needed to adapt to a different clinical treatment environment, especially when providing nursing care for female patients. Some participants described their mixed emotions about caring for female patients and how they learn to adapt, step by step (Blair, 2016; Smith et al., 2020):

Um, you know, most of my patients are females. Maybe once every two or three months, I have a female patient that would not want a male nurse to take care of her. Before providing nursing care, I always told them “Please don't be nervous. I'm a nurse. Just performing the duties of a nurse.” So, I just maintain my professionalism, and I don't make any jokes about what I'm doing… (Participant Legend, Blair, 2016, p. 114)

## 5 DISCUSSION

The results suggest that value in nursing is a predominant theme throughout a nursing career engaged in by men. Men in nursing perceived happiness, well‐being, achievability and spiritual satisfaction due to alleviating the suffering of patients, rapid recovery of patients, praying with patients and providing them with confidence to overcome illness. These findings revealed that men in nursing had an inherent desire to care for others and to be involved with helping others. As demonstrated in Mooney et al. (2008) and Wu et al.'s (2015) studies, nursing is seen as a noble and worthwhile profession by most nursing students and clinical nurses. This also explains the altruistic characteristics related to professional values which become more salient to healthcare professionals (Hayes & Shakya, 2013; Mathauer & Imhoff, 2006). As van der Wath and van Wyk (2020) reported, nurses experienced gratitude that nursing provided them with opportunities to help others. They said valuable and meaningful when they practised altruism. Thus, altruism should be encouraged and developed to include men and women nurses as a means to improve the experience of those engaged in the nursing profession.

The participants acknowledged the healing power of spirituality and affirmed the significant influence of spirituality on their careers and on alleviating the sickness of patients. They claimed that the spirit is a source of power to overcome the workload in their careers. This is a unique finding in our study about men in nursing. It is possible that this finding is not specific to men in nursing but also women. Although nurses play a crucial role in providing individualized spiritual care to patients, spirituality is a complex concept, and it is unclear when it comes to the difference between men and women nurses about providing spiritual care, but the difference does not preclude nursing care provided by men. Thus, it should be considered to further research.

The participants also noted that the nursing profession provides them with more job opportunities, job security, economic stability and job flexibility. As demonstrated by Zamanzadeh et al. (2013), these are pivotal influencing factors for men to willingly choose nursing. Although this finding has been confirmed in many previous studies, it is not specific to men in nursing but also women. Thus, it can be seen that nursing is a profession with vast job opportunities and job flexibility, which further leads to job security and economic stability because nurses can advance in their careers, work more than one job or do extra shifts that lead to increased income. According to Lent et al. (2000) when a person decides to pursue a career in nursing, the expected outcomes and goals are considered to be one of the most important influencing factors. Nursing provided men with job opportunities, financial stability and job flexibility; thus, fulfilling their expected outcomes and goals.

In this study, men experienced different degrees of gender disadvantages in the nursing profession. This finding is similar to that of previous studies (Stanley et al., 2016; Zhang & Liu, 2016). However, this indirectly implies that nursing would not be a suitable profession for men. This gender stereotyping forces men to re‐check whether the nursing profession is suitable for them. Thus, it will lead men to consider choosing non‐nursing professions in the future which is a possible reason for the persistently low numbers of men in nursing. Meanwhile, this study confirmed findings in the literature about gender‐based stereotypes, discrimination and isolation affecting men in nursing (Adeyemi‐Adelanwa et al., 2016; Cheng et al., 2018; Zhang & Tu, 2020). The persistence of these stereotypes implies that discrimination and isolation will not end easily and may continue to hamper men. In addition, their physical strength was an apparent gender stereotype especially when patients needed to be moved. More interestingly, because of gender‐based stereotypes, the participants reported that being men in nursing had a negative impact on their marriage prospects. This finding was confirmed by previous studies (Saleh et al., 2020; Zhang & Tu, 2020) in Chinese and Arab contexts as well.

Despite the gender‐based stereotypes in the nursing profession mentioned above, men also experienced some gender advantages, for instance, super‐resilience, endurance and promotion prospects. In addition, they were considered to be better at dealing with machines than women. This is in line with a previous study conducted in Macau (Mao et al., 2021). This “usefulness” is further evidence of gender advantage. Therefore, the gender advantage may let men circumvent the barriers and play to their strengths in the nursing profession. Another gender advantage can be described as a “glass escalator” mentioned by Williams (1992). The glass escalator is a term that refers to men having an advantage in terms of opportunity, promotion and salary in that they can achieve advancement or promotion faster in a given field than women. Men in nursing received gender advantages in terms of career opportunities and promotions, and they were expected to take on more responsibilities (Zhang & Tu, 2020). It is possible that men in nursing are more visible in a largely women‐dominated profession. Overall, gender advantages led to men being readily offered positions in the nursing profession and with more benefits.

Since men in nursing are skilful with their craft, are polite and courteous, have good professional conduct and cooperate in work groups and provide a comforting atmosphere when taking care of their patients, they were welcomed by patients and women nurse peers. This finding is consistent with previous studies (Budu et al., 2019; Gedzyk‐Nieman & Svoboda, 2019; Mao et al., 2021), which demonstrate that men's performances were appreciated in the nursing profession. It shows that men in nursing are using their concrete actions to indirectly influence patients and women peers to change the social perspective and create gender equality in the nursing profession. However, the influence of women nurse peers on men's success was not explicit in the nursing profession. Interestingly, there was a dearth of research studies focussing on the family support of men in nursing. Studies on women in nursing were more directed towards how to influence men's successes, making studies about family support of men in nursing more significant and desirable as future research studies.

As a minority group in the nursing profession, the participants noted that there is a lack of a positive appearance of men in public media, and there is a limited number of role models of men in the nursing profession or nursing schools. These findings have been reported in previous studies (Barrett‐Landau & Henle, 2014; Powers et al., 2018).

It is recommended that men in nursing should be presenting themselves to the public to build a role model of men in the healthcare delivery system to decrease social stigmas and promote acceptance of gender diversity in the nursing profession. Men in nursing need to attend various events as often as possible to present a good impression in public, for instance at nursing forums, nursing seminars and nursing ward rounds. Department meetings and when patients are being discharged are situations in which men in nursing will be easily noticed by patients and their families, women nurse peers and other healthcare professionals. Herakova (2012) claimed that the image of role models is formed by fulfilling cultural expectations and emotional support. As previous studies reported, men in nursing provided better nursing care to patients, which may lead men in nursing to become more visible and finally enable them to stand out (Smith et al., 2020; Zhang & Tu, 2020).

Besides, men in nursing have struggled to adapt themselves professionally in a women‐dominated field through strategies such as empowering themselves through self‐development, getting along well with women nurse peers, and providing a good quality of nursing care to female patients, thereby expanding their influence in the nursing profession. As Zamanzadeh et al. (2013) pointed out, caring is not an inherently feminine trait and men in nursing can be as caring as women nurses. If everyone believes that a nurse is only a nurse and care is only care, perhaps the public will observe less difference between men and women nurses' caring. Thus, actively adapting themselves to the nursing care environment is essential for men in nursing. If this is a given, men in nursing deserve the necessary support to help them to better adapt to the nursing profession.

### 5.1 Limitations

This study is the first one focussing on a qualitative systematic review of the lived experience of men in nursing. Thus, this systematic review has some limitations worth noting. Firstly, this updated review of the lived experience of men in nursing focussed on predominantly English language publications. Secondly, only six articles were included in this study, which may result in a less fully revealed lived experience of men in nursing. Finally, this study focussed on the lived experience of men in nursing who had worked in the nursing profession for at least 1 year, which is not representative of all men in nursing. Thus, future research could consider the lived experience of novice men in the nursing profession as well.

## 6 CONCLUSION

The findings of this systematic review provide a better understanding of the lived experiences of men in nursing. They allow nursing managers, educators and clinical practitioners to be more aware of the feelings and perceptions of men in the nursing profession. The findings help to remove gender barriers in nursing, which will facilitate recruitment and retention of men in the nursing profession. In addition, the issues related to gender‐based stereotypes should be addressed for improving the value of men in the nursing career. Women nurses and other healthcare professionals should not unduly exploit men for physical strength and limit their patient care assignments or experiences. One interesting finding in the study is that men in nursing play crucial roles in providing individualized spiritual care to patients. However, it is unclear whether or not there are differences between men and women nurses in providing spiritual care. Thus, more studies should be considered in future research.

## AUTHOR CONTRIBUTIONS

Conception and design: Xiaochen Lyu and Thitinut Akkadechanunt; Data collection and analysis: Xiaochen Lyu, Thitinut Akkadechanunt, Pratum Soivong and Ratanawadee Chontawan; Manuscript preparation: Xiaochen Lyu, Thitinut Akkadechanunt, Phanida Juntasopeepun and Ratanawadee Chontawan.

## CONFLICT OF INTEREST

The authors have no conflict of interest to declare.

## Data Availability

The data that support the findings of this study are available from the corresponding author upon reasonable request.
